# Association of environmental surface contamination with hand hygiene and infections in nursing homes: a prospective cohort study

**DOI:** 10.1016/j.infpip.2021.100129

**Published:** 2021-02-28

**Authors:** G.R. Teesing, M. de Graaf, M. Petrignani, V. Erasmus, C.H.W. Klaassen, C.M.E. Schapendonk, A. Verduijn-Leenman, J.M.G.A. Schols, M.C. Vos, M.P.G. Koopmans, J.H. Richardus, H. Voeten

**Affiliations:** a Department of Public Health, Erasmus MC, University Medical Centre Rotterdam, Rotterdam, the Netherlands; b Municipal Public Health Service Rotterdam-Rijnmond, Rotterdam, the Netherlands; c Viroscience Department, Erasmus MC, University Medical Centre Rotterdam, Rotterdam, the Netherlands; d Municipal Public Health Service Amsterdam, Amsterdam, the Netherlands; e Department of Medical Microbiology and Infectious Diseases, Erasmus MC, University Medical Centre Rotterdam, Rotterdam, the Netherlands; f Pieter van Foreest (retired), Delft, the Netherlands; g Department of Health Services Research, CAPHRI, Maastricht University, Maastricht, the Netherlands

**Keywords:** Healthcare-associated infection, Hand hygiene, Micro-organism, Environmental surface sampling

## Abstract

**Background:**

Little is known about the presence of infections in nursing home residents, the causative micro-organisms, how hand hygiene (HH) influences the presence of infections in residents, and the extent to which environmental contamination is associated with the incidence of infection among residents.

**Aims:**

To establish if environmental contamination can be used as an indicator for HH compliance, and if environmental contamination is associated with the incidence of infection.

**Methods:**

Environmental surface samples (ESS) were collected in an exploratory study as part of a HH intervention in 60 nursing homes. ESS results from three distinct surfaces (nurses' station, communal toilet and residents' shared living area) were compared with nurses' HH compliance and the incidence of infection among residents. Real-time polymerase chain reaction assays were used to detect norovirus genogroup I and II, rhinovirus and *Escherichia coli*. HH compliance was measured by direct observation. The incidence of infection was registered weekly.

**Findings:**

Rhinovirus (nurses' station: 41%; toilet: 14%; living area: 29%), norovirus (nurses' station: 18%; toilet: 12%; living area: 16%) and *E. coli* (nurses' station: 14%; toilet: 58%; living area: 54%) were detected. No significant (*P*<0.05) associations were found between HH compliance and the presence of micro-organisms. An association was found between *E. coli* contamination and the incidence of disease in general (*P*=0.04). No other associations were found between micro-organisms and the incidence of disease.

**Conclusion:**

Rhinovirus, norovirus and *E. coli* were detected on surfaces in nursing homes. No convincing associations were found between environmental contamination and HH compliance or the incidence of disease. This study provides reference data about surface contamination.

## Introduction

Healthcare-associated infections (HAIs) are a major cause of morbidity and mortality in nursing homes (NHs). The European Centre for Disease Prevention and Control estimates a prevalence of 38 HAIs per 1000 resident-days in long-term health care, with the most prevalent being respiratory infection, urinary tract infection (UTI) and skin/soft tissue infection [1,2]. Infections can be endogenous or exogenous, and increased compliance with hand hygiene (HH) can decrease the exogenous infection rate [3]. Poor HH compliance by healthcare workers can result in higher rates of infection through the transmission of micro-organisms from an infected resident or healthcare worker to another resident through either direct contact or fomite transmission. HAI has also been shown to be associated with the complexity of care, resident characteristics, duration of contact, number of contacts and type of contact [4,5].

The evaluation of HH compliance is challenging. Direct observation is costly and can be affected by the Hawthorne effect or observer bias, and automated HH monitoring systems do not register all HH opportunities [6,7].

Disease monitoring can also be challenging in NHs. In the Netherlands, few NH organizations perform disease surveillance. This is contrary to hospitals, where infection surveillance is part of a quality system with dedicated staff to register illness and perform sampling, and diagnostics are used to determine causative micro-organisms.

Environmental surface sampling (ESS) is commonly used in the control of food safety or veterinary infections to detect environmental contamination after an outbreak, but this method has not, to the authors' knowledge, been used to monitor HH compliance of nurses or as a proxy for infections among residents in NHs [8]. ESS is an objective measurement tool that is not dependent on the observations of either nurses or observers [9,10]. The challenges in evaluating HH compliance and disease surveillance mentioned led the authors to execute exploratory research to establish if environmental contamination can be used as an indicator for HH compliance; and if environmental contamination is associated with the incidence of infection.

## Methods

### Study design

This cohort study explored the presence of indicator micro-organisms in the environment, and associations with HH compliance of nurses and the incidence of infection among residents (as measured prospectively in the HANDSOME study) [11]. HANDSOME, a cluster randomized controlled trial in publicly funded Dutch NHs, determined the increase in HH compliance among nurses after a multi-modal HH intervention. The NHs in the intervention arm received the intervention and those in the control arm did not receive any intervention. The multi-modal intervention targeted NH policy changes by auditing personal hygiene rules as well as available HH materials, and targeted behaviour of nurses through e-learning, three live lessons, posters, and a photo competition. Data were collected between October 2016 and October 2017. Eighteen NH organizations committed three or four NHs to the study. All NHs provided psychogeriatric and/or somatic care to geriatric residents. The protocol and HH compliance results are described elsewhere [11,12]. The study population was diverse in terms of the size of the organization, urbanization, type of care, and staff-to-resident ratio. Ethical approval for the study was waived by the Medical Ethics Review Committee of Erasmus MC (Ref. 58158) as the residents were not subjected to sampling, treatment, or behaviour rules.

### Hand hygiene compliance

HH compliance was measured through unobtrusive direct observation. HH compliance was defined as the use of alcohol-based hand rub or soap, water and a paper towel. NHs were observed from 8 am to 12.30 pm in October 2016 (baseline), February 2017 (during the intervention) and May 2017 (after the intervention). There was originally a third arm in the study, but this was discontinued because six NHs in this arm were not able to implement the intervention and observers were not available for certain observation periods. As there were concurrent baseline measurements from this third arm, these were included in the present study. HH opportunities were defined according to the World Health Organization's ‘Five Moments’, namely: before touching a resident, before a clean/aseptic task, after body fluid exposure, after touching a resident, and after touching a resident's surroundings [13]. As this study was performed in NHs, the surroundings were defined as the resident's room or that portion of the room that belonged to the resident. HH was registered by trained research assistants in a novel app [11]. In total, 426 nurse observations and 5200 HH opportunities were included in this study. Sixty NHs were included in the trial, representing a total of 3284 beds. Of these, 85% participated through May 2017 (51/60 NHs) and 15% left the study prematurely for various reasons. Aggregated HH compliance at baseline was 11% (range 1–26%). This increased to 27% (range 7–53%) in May 2017 [12].

### Environmental sampling in nursing homes

The presence of rhinovirus (a common respiratory virus), norovirus (a common cause of non-bacterial gastroenteritis) and *Escherichia coli* (an indicator of faecal contamination and general hygiene) were examined [9,[14], [15], [16]]. Rhinovirus was chosen as this is one of the most common (9%) causes of respiratory infection in institutionalized elderly people [10]. Norovirus was chosen based on studies in healthcare facilities which found that norovirus is frequently detected and a leading cause of HAI-associated death in individuals aged >65 years [17].

Environmental swab samples were collected by trained research assistants at the end of each HH observation session. Sterile, ready-to-use wipes, prewetted with 10 mL of Ringer's solution (Sodibox, Névez, France), were used for swabbing following the protocol of the Food Safety Authority [18]. Three high-contact surfaces for HH were targeted to determine circulation of the targeted micro-organisms in the facility: the computer keyboard and mouse at the nurses' station (used solely by staff), a table in a communal living area (used primarily by residents), and the toilet flushing knob and toilet seat of a communal toilet (used primarily by residents). It was assumed that the keyboard and mouse would give an indication of micro-organism contamination by nurses, and that the table in the living room would primarily give an indication of micro-organism contamination by residents.

### Processing of swab samples

Wipes were placed in a 50-mL tube with sterile forceps for each sample, after which 15 mL of lysis buffer was added to each tube. Nucleic acid was isolated using the Boom method [18]. Real-time polymerase chain reaction assays were used for the detection of norovirus genogroup I and II, rhinovirus and *E. coli*. The viral micro-organisms were detected by primers and probes used in the routine molecular viral diagnostics setting of Erasmus Medical Centre, as described previously [[19], [20], [21]]. *E. coli* was detected using primers and probes as described by Pavlovic *et al.* [22]. Most samples were positive for *E. coli* to some extent, and in some cases had very high cycle threshold (Ct) values; for these analyses, samples with an arbitrary cut-off of Ct >35 for *E. coli* were considered negative.

### Incidence of infection

Infections of residents over a 7-week period were considered: 3 weeks before ESS, the week of ESS, and 3 weeks after ESS. This study therefore considered the incubation period (1–3 days), at least one serial interval (1–3 days) and the shedding period (rhinovirus: 1–2 weeks, norovirus: 3 weeks) of the included micro-organisms to detect circulation [[23], [24], [25]]. This study also considered that norovirus survives and remains detectable on hard surfaces for days or weeks [26]. Infection registration started during or after the first round of ESS; the baseline had a maximum registration period of 4 weeks.

Each NH unit had a self-designated staff member (nurse, team leader, or geriatrician) who recorded the weekly incidence of gastroenteritis, influenza-like illness (ILI), suspected pneumonia, UTI, and meticillin-resistant *Staphylococcus aureus* (MRSA) on a uniform form. The McGeer criteria were used to define illnesses, and MRSA was laboratory confirmed [27]. Data on the incidence of infection were anonymized and aggregated.

### Analysis

The presence of rhinovirus, norovirus, and *E. coli* was noted and expressed as a percentage of NHs that had the micro-organism per observation round. HH compliance was calculated by dividing the number of compliant HH opportunities by the total number of HH opportunities, and expressed as a percentage [12]. A multi-level analysis was performed subsequently to determine if the presence of a micro-organism in one of the three sampling locations (nurses' station, toilet or living area) was an inverse predictor of HH compliance. All multi-level analyses in this paper controlled for: (i) the clustering of observations within NHs; (ii) period; and (iii) whether the NH received the intervention.

Next, the association between the incidence of HAI and positive environmental samples was examined. The 7-week period of HAI registration per NH per round was aggregated, and multi-level analyses were used to investigate if the presence of each individual micro-organism was a predictor of infection in general, and whether the presence of norovirus was associated with gastroenteritis.

Next, the association between background variables and surface contamination was explored using a multi-level model. Background variables were included if data were available from at least 75% of the NHs. The following background variables were included: number of beds in the unit; complexity of care as determined by a care indication (‘zorgzwaartepakket’); number of residents per bathroom; presence of a tap in every bedroom; presence of a tap in every shared living area; whether the healthcare workers worked on one or multiple wards; whether it is standard practice that residents are informed about HH; the percentage of residents that wash themselves; how often the residents' rooms are cleaned; how often the bathrooms/toilets are cleaned; whether HH reminders were hung somewhere; the number of nurses per beds in the NH; whether alcohol-based hand rub is available in all bedrooms; and the percentage of residents that were able to go to the toilet without assistance. Association of these background variables with any surface contamination (either norovirus, rhinovirus and/or *E. coli*) at any of the locations (living room, toilet and/or nurses' station) was examined. All analyses were performed using SPSS Version 25 (IBM Corp, Armonk, NY, USA).

## Results

### Detection of environmental contamination

**Figure 1:**
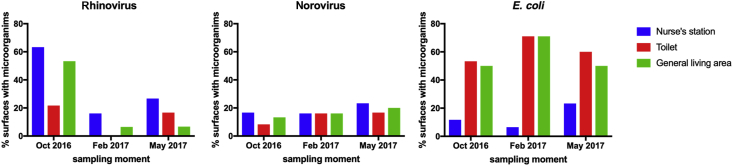
Percentage of nursing homes where selected micro-organisms were found on environmental surfaces over three periods (N=60, 31 and 30 nursing homes per sampling moment, respectively). Blue bars, nurses' station; red bars, toilet; green bars, general living area. E. coli, Escherichia coli.

Positive samples were detected (*N*=121 per surface) for rhinovirus (nurses' station: 41%; toilet: 14%; living area: 29%), norovirus (nurses' station: 18%; toilet: 12%; living area: 16%) and *E. coli* (nurses' station: 14%; toilet: 58%; living area: 54%) (Figure 1). Generally, more positive rhinovirus samples were found at the nurses' station and in the general living area compared with the toilet. In contrast, there were no clear differences in the presence of norovirus RNA in all three sampling areas, although the average level of virus contamination per positive sample was highest for the toilet (data not shown). The percentage of positive *E. coli* samples was lowest for the nurses' station. Only rhinovirus presented a clear pattern over time, with a reduction in total positive samples at the second and third timepoints.

### Association between environmental contamination and hand hygiene compliance

In order to assess the association between HH compliance and environmental contamination, the authors tested for significant differences in average HH compliance between NHs where micro-organisms were present and NHs where micro-organisms were not present. Average HH compliance ranged from 12% to 20% when a micro-organism was present and from 16% to 21% when a micro-organism was not present (Table I). In the multi-level regression model, no significant (*P*<0.05) associations between HH compliance and the presence of a micro-organism were found, although there was a weak association (*P*=0.07) between rhinovirus in the living area and HH.

**Table I:** Average hand hygiene compliance per nursing home (NH), comparing NHs where micro-organisms were present on surfaces and NHs where micro-organisms were not present on surfaces, to test the association between environmental contamination and hand hygiene compliance (*n*=121 NH observation-days in 60 NHs, *n*=8928 hand hygiene observations).

Tested micro-organism	Sampling location	Micro-organism present		Micro-organism not present		*P*-value^a^
		Average hand hygiene compliance (%)	Percentage of NHs with micro-organism (%)	Average hand hygiene compliance (%)	Percentage of NHs without micro-organism (%)	
Rhinovirus	Nurses' station	14	43	20	58	0.79
	Toilet	16	15	18	85	0.26
	Living area	12	30	21	70	0.07
Norovirus	Nurses' station	16	18	18	82	0.82
	Toilet	18	13	17	88	0.66
	Living area	20	15	17	85	0.87
*Escherichia coli*	Nurses' station	18	13	17	87	0.87
	Toilet	18	60	16	40	0.58
	Living area	18	56	17	44	0.80

a Controlled for the clustering of observations within NHs, period, and whether the NH received the intervention in a multi-level regression model.

### Association between environmental contamination and incidence of infection

First, the incidence rates of gastroenteritis, ILI, pneumonia, UTI, MRSA and a combination of gastroenteritis, ILI and pneumonia, per 1000 resident-days in each period, were examined (Table II). Average incidence rates per period were low; the highest mean incidence was for UTI (1.40–2.07 per 1000 resident-days). The lowest incidence was for MRSA, with 0–0.07 incidents per 1000 resident-days per round. When gastroenteritis, ILI and pneumonia-like illnesses were combined, the range was 0–11.90 cases per 1000 resident-days per NH for all periods, with an overall average per period ranging from 1.27 to 2.71 per 1000 resident-days. Considerable differences were evident between NHs regarding numbers of HAIs reported. For the periods observed, 7% of the NHs reported no infections.

**Table II:** Infections during three sampling rounds. For every round, 7 weeks from the infection registry were included (3 weeks before sampling, the week of sampling and 3 weeks after sampling). In October 2016, registered infections started the week of environmental sampling (*n*=116 nursing home observation-days^a^ in 60 nursing homes with *N*=5200/1852/1876 observations per round).

Sampling round	Infections per 1000 resident-days	Mean	Median	Standard deviation	Minimum	Maximum
October 2016 (*N*=55)	Gastroenteritis	1.20	0.00	2.20	0.00	10.27
	ILI	0.84	0.00	1.71	0.00	8.93
	Pneumonia	0.66	0.51	0.76	0.00	2.93
	Urinary tract infection	2.07	1.59	2.09	0.00	10.20
	MRSA	0.02	0.00	0.11	0.00	0.64
	Combination of gastroenteritis, ILI and pneumonia	2.71	1.76	2.90	0.00	11.90
February 2017 *(N*=31)	Gastroenteritis	0.95	0.00	1.80	0.00	7.02
	ILI	1.15	0.00	1.95	0.00	8.63
	Pneumonia	0.50	0.42	0.52	0.00	1.83
	Urinary tract infection	1.40	1.12	1.34	0.00	5.44
	MRSA	0.00	0.00	0.00	0.00	0.00
	Combination of gastroenteritis, ILI and pneumonia	2.60	1.24	2.82	0.00	11.38
May 2017 *(N*=30)	Gastroenteritis	0.45	0.00	1.07	0.00	4.25
	ILI	0.30	0.00	0.99	0.00	5.10
	Pneumonia	0.51	0.45	0.59	0.00	2.63
	Urinary tract infection	1.59	1.34	1.28	0.00	5.10
	MRSA	0.07	0.00	0.33	0.00	1.81
	Combination of gastroenteritis, ILI and pneumonia	1.27	0.72	1.86	0.00	8.50

ILI, influenza-like illness; MRSA, meticillin-resistant *Staphylococcus aureus*.

a Five nursing homes started their infection registry ≥4 weeks after the first observation.

Next, the authors investigated whether the indicator micro-organisms were associated with infectious disease. When all variables were tested in the multi-level model, only *E. coli* contamination of the toilet was significantly associated with the incidence of disease in general (*P*=0.04) (Table III). A weak association (*P*=0.06) was found between norovirus at the toilet and gastroenteritis. None of the other micro-organisms on any of the three surfaces were associated with the incidence of disease.

**Table III:** Association between the presence of surface micro-organisms in nursing homes (NHs) and the incidence of disease (*N*=116 NH observation-days in 60 NHs, 7-week illness registration per period for three periods).

Micro-organism	Place found	Infection^a^	Micro-organism present		Micro-organism not present		*P*-value^b^
			Incidence of disease per 1000 resident-days	Percentage of NHs homes with micro-organism	Incidence of disease per 1000 resident-days	Percentage of NHs with micro-organism	
Rhinovirus	Nurses' station	I/G/P	2.35	41	2.27	59	0.88
	Toilet	I/G/P	1.92	15	2.37	85	0.47
	Living area	I/G/P	2.49	29	2.23	71	0.70
Norovirus	Nurses' station	I/G/P	2.78	19	2.19	81	0.65
	Toilet	I/G/P	2.75	13	2.24	87	0.44
	Living area	I/G/P	1.67	16	2.43	84	0.41
*Escherichia coli*	Nurses' station	I/G/P	1.92	14	2.36	86	0.48
	Toilet	I/G/P	2.74	59	1.68	41	0.04
	Living area	I/G/P	2.55	55	2.01	45	0.82
Norovirus	Nurses' station	Gastroenteritis	1.63	19	0.78	81	0.17
	Toilet	Gastroenteritis	1.71	13	0.83	87	0.06
	Living area	Gastroenteritis	0.28	16	1.07	84	0.11

a I/G/P: either influenza-like illness, gastroenteritis or pneumonia.

b Controlled for the clustering of observations within NHs, period, and if the NH received the intervention in a multi-level analysis.

### Association between environmental contamination and background variables

Finally, significant associations between surface contamination and background variables of NHs were investigated. After Bonferroni's correction to account for testing 79 possible associations (α=0.0006), none of the variables were significantly associated with ESS (Table S1, see online supplementary material).

## Discussion

This exploratory study in NHs detected rhinovirus (41% at the nurses' station, 14% in the toilet and 29% in the living area), norovirus (18% at the nurses' station, 12% in the toilet and 16% in the living area) and *E. coli* (14% at the nurses' station, 58% in the toilet and 54% in the living area). No significant (*P*<0.05) associations were found between HH compliance and the presence of a micro-organism, although there was a weak association (*P*=0.07) between rhinovirus in the living area and HH. With regard to environmental contamination and HAI occurrence, there was an association between *E. coli* contamination and the incidence of disease in general (*P*=0.04), and a weak association (*P*=0.06) between norovirus and gastroenteritis. None of the other micro-organisms on any of the three surfaces were associated with the incidence of disease.

Other studies have also detected the micro-organisms selected in this study on different types of surfaces. Shortly after or during outbreaks, levels of norovirus contamination on surfaces in catering companies were up to 40%, while in non-outbreak-related establishments, only 2% of the surfaces tested gave positive results for norovirus [8]. Similar observations were reported in other settings such as military garrisons, cruise ships and long-term care facilities [28,29]. Besides *E. coli*, faecal contamination on surfaces has also been studied by testing CrAssphage [29].

Several reasons could explain why little to no relationship was found between ESS results and HH compliance. Firstly, the differences in HH compliance levels between NHs were small, impacting the power of the analyses. Secondly, norovirus and *E. coli* may be more difficult to eliminate when using alcohol-based hand rub than other micro-organisms [30,31]. In this study, 51% of HH compliance was achieved with alcohol-based hand rub (data not shown). There is evidence that alcohol-based hand rub is effective for eliminating rhinovirus [32,33]. This may explain why there was some evidence of a reduction in rhinovirus when HH compliance was higher. Thirdly, viruses can also spread through droplets and aerosols (i.e. through coughing and vomiting). These droplets and aerosols would fall on surfaces and thus be detectable but unrelated to HH. This is particularly the case for rhinovirus, and could occur for norovirus but not for *E. coli* [34,35]. Fourthly, contamination of a surface, such as a computer keyboard, implies that at least one person had poor HH, but does not indicate average HH compliance. Finally, other unstudied factors which were not taken into account may also influence the association between ESS and HH compliance levels, such as how many hours/days there were betweeen cleaning surfaces and taking environmental samples, the quality of cleaning of the different surfaces, HH of nursing assistants, and HH of residents.

Recognizing disease can be challenging in NH residents as their symptoms can be more subtle and differ from those in younger populations. Taking samples for diagnostic tests can also be more challenging in an elderly population with psychogeriatric disorders, and therefore difficult to justify ethically when research is the main goal. ESS may help to gain insight into which diseases are circulating in the environment. However, the relationship between ESS results and HAI (per 1000 resident-days) is complicated for various reasons. For example, a single ill or infectious person could cause positive ESS results. It may therefore be better to use a dichotomous variable (some/no illness in the NH) to understand the association between the presence of infections and positive samples, rather than the number of infections per 1000 resident-days. Also, if surfaces were cleaned immediately before the samples were taken, this may have eliminated potential positive samples and therefore weakened the relationship between HAI and positive ESS. A third issue is that this study included a standard instrument for HAI reporting in NHs which did not include the common cold. Consequently, a potential association between HAI and rhinovirus was missed. There was a significant association between *E. coli* and HAI. One possible explanation for this is that less hygienic NHs are more likely to experience HAIs [36].

The detection of micro-organisms was also assumed to be affected by seasonal differences in the prevalence of viruses. For example, rhinovirus circulates throughout the year, but generally has slightly more infections in the autumn and fewer infections in the summer [37]. Norovirus also presents seasonal differences, with most outbreaks occurring in the winter [38]. Thus, during the third period (May 2007), the prevalence of the indicator viruses could be lower than in the first two periods, potentially affecting the outcomes.

## Conclusions and recommendations

Further exploration of ESS is recommended, where detection in the environment is followed by sampling residents to further validate this method. Any future study including rhinovirus should incorporate surveillance of the common cold to enable better association of the observed illness and the target micro-organism. Another suggestion is that similar studies should be performed within a limited time frame when the illnesses caused by these micro-organisms are most prevalent.

To conclude, the authors were able to detect rhinovirus, norovirus and *E coli* on surfaces in NHs. No convincing associations were found between environmental contamination and HH compliance or the incidence of disease. This study provides reference data on surface contamination.

## CRediT author statement

**G.R. Teesing:** Methodology, Investigation, Formal analysis, Data curation, Writing- Original draft. **M. de Graaf:** Methodology, Investigation, Formal analysis, Writing- Original draft. **M. Petrignani:** Conceptualization, Methodology, Writing-reviewing and editing. **V. Erasmus:** Conceptualization, Methodology, Writing-reviewing and editing. **C.H.W. Klaassen:** Investigation, Validation, Writing-reviewing and editing. **C.M.E. Schapendonk:** Investigation, Validation, Writing-reviewing and editing. **A. Verduijn-Leenman:** Conceptualization, Methodology, Writing-reviewing and editing. **J.M.G.A. Schols:** Conceptualization, Methodology, Writing-reviewing and editing. **M.C. Vos:** Conceptualization, Methodology, Writing-reviewing and editing. **M.P.G. Koopmans:** Conceptualization, Methodology, Writing-reviewing and editing. **J.H. Richardus:** Conceptualization, Methodology, Writing-reviewing and editing, Supervision, Funding acquisition. **H. Voeten:** Conceptualization, Methodology, Writing-reviewing and editing, Supervision, Funding acquisition.

## Conflict of interest statement

None declared.

## Funding sources

This study was funded by the Netherlands Organization for Health Research and Development (ZonMw) (Grant No. NL50-53000-98-151). MdG was funded through the European Community's Horizon 2020 Research and Innovation Programme under the COMPARE Project (Grant No. 643476). Non-financial support was received from Essity during the study period. The full trial protocol can be accessed at the Netherlands Trial Register, Trial NL6049 (NTR6188): https://www.trialregister.nl/trial/6049.
