# Supplementary material for: Association of environmental surface contamination with hand hygiene and infections in nursing homes: a prospective cohort study
Source: Infect Prev Pract. 2021 Feb 28;3(2):100129. doi: 10.1016/j.infpip.2021.100129 (PMC8335935; doi:10.1016/j.infpip.2021.100129)
Supplement: Multimedia component 1 [file mmc1.docx]

**Table S1**

Background variables and surface contamination^a^

| Background variable | | | Percentage of NHs (*N*=60) | Rhinovirus, norovirus or *E. coli* in living room, *P*-value (*N*=93/121) | Rhinovirus or norovirus at toilet, *P*-value (*N*=28/121) | Rhinovirus, norovirus or *E. coli* at nurses’ station, *P*-value (*N*=69/121) | Norovirus anywhere, *P*-value (*N*= 38/121) | Rhinovirus anywhere, *P*-value (*N*=66/121) | *E. coli* in living room or at nurse's station, *P*-value (*N*=71/121) |
| --- | --- | --- | --- | --- | --- | --- | --- | --- | --- |
| Number of beds in units | | |  | 0.65 | 0.70 | 0.45 | 0.35 | 0.38 | 0.78 |
|  | <45 | | 32% |  |  |  |  |  |  |
|  | 45–59 | | 23% |  |  |  |  |  |  |
|  | ≥60 | | 45% |  |  |  |  |  |  |
| Complexity of care | | |  | 0.79 | 0.02 | 0.80 | 0.75 | 0.91 | 0.98 |
|  | Only high complexity | | 73% |  |  |  |  |  |  |
|  | All level of care | | 25% |  |  |  |  |  |  |
|  | Missing | | 2% |  |  |  |  |  |  |
| Number of residents per bathroom | | |  |  | 0.08 |  | 0.92 | 0.65 |  |
|  | ≥2 | | 70% |  |  |  |  |  |  |
|  | 1 | | 30% |  |  |  |  |  |  |
| Tap in every bedroom | | |  | 0.33 | 0.93 | 0.26 | 0.07 | 0.73 | 0.45 |
|  | Yes | | 63% |  |  |  |  |  |  |
|  | No | | 32% |  |  |  |  |  |  |
|  | Missing | | 5% |  |  |  |  |  |  |
| Tap in every shared living area | | |  | 0.47 |  |  | 0.69 | 0.26 | 0.66 |
|  | | Yes | 85% |  |  |  |  |  |  |
|  | | No | 10% |  |  |  |  |  |  |
|  | | Missing | 5% |  |  |  |  |  |  |
| Healthcare workers work in a single unit or multiple units | | |  | 0.85 | 0.66 | 0.10 | 0.70 | 0.46 | 0.52 |
|  | Single unit | | 88% |  |  |  |  |  |  |
|  | Multiple units | | 10% |  |  |  |  |  |  |
|  | Missing | | 2% |  |  |  |  |  |  |
| Standard practice is that residents are informed about good hand hygiene | | |  | 0.36 | 0.67 | 0.76 | 0.27 | 0.71 | 0.66 |
|  | | Yes | 17% |  |  |  |  |  |  |
|  | | No | 82% |  |  |  |  |  |  |
|  | | Missing | 2% |  |  |  |  |  |  |
| Percentage of residents that wash themselves | | |  | 0.21 | 0.18 | 0.27 | 0.77 | 0.90 | 0.23 |
|  | | ≤10% | 85% |  |  |  |  |  |  |
|  | | >10–25% | 15% |  |  |  |  |  |  |
| How often are residents’ rooms cleaned? | | |  |  |  |  | 0.85 | 0.13 | 0.68 |
|  | | More than once per week | 25% |  |  |  |  |  |  |
|  | | Weekly | 53% |  |  |  |  |  |  |
|  | | Missing | 22% |  |  |  |  |  |  |
| How often are bathrooms and toilets cleaned? | | |  |  | 0.52 |  | 0.86 | 0.57 | 0.68 |
|  | | More than once per week | 47% |  |  |  |  |  |  |
|  | | Weekly | 33% |  |  |  |  |  |  |
|  | | Missing | 20% |  |  |  |  |  |  |
| HH reminders hung somewhere | | |  | 0.70 | 0.62 | 0.71 | 0.64 | 0.87 | 0.21 |
|  | | Yes | 55% |  |  |  |  |  |  |
|  | | No | 25% |  |  |  |  |  |  |
|  | | Missing | 20% |  |  |  |  |  |  |
| Number of nurses per resident | | |  | 0.33 | 0.04 | 0.83 | 0.56 | 0.96 | 0.55 |
|  | | <1 | 67% |  |  |  |  |  |  |
|  | | >1 | 33% |  |  |  |  |  |  |
| Hand sanitizer is available in all bedrooms | | |  | 0.67 | 0.89 | 0.38 | 0.03 | 0.71 | 0.60 |
|  | | Yes | 40% |  |  |  |  |  |  |
|  | | No | 53% |  |  |  |  |  |  |
|  | | Missing | 7% |  |  |  |  |  |  |
| Percentage of residents that can go to the toilet without assistance | | |  | 0.65 | 0.93 | 0.41 | 0.33 | 0.89 | 0.18 |
|  | | <20% | 55% |  |  |  |  |  |  |
|  | | ≥20% | 45% |  |  |  |  |  |  |
| NH, nursing home; HH, hand hygiene; *E. coli*, *Escherichia coli.*  ^a^Controlled for the clustering of observations within NHs, period, and if the NH received the intervention in a multi-level regression model. Bonferroni’s correction was used to account for 79 possible correlations. Therefore, the *P*-value should be <0.0006 for an association to be considered significant. | | | | | | | | | |
